# Extracellular Vesicles Taken up by Astrocytes

**DOI:** 10.3390/ijms221910553

**Published:** 2021-09-29

**Authors:** Ari Ogaki, Yuji Ikegaya, Ryuta Koyama

**Affiliations:** 1 Laboratory of Chemical Pharmacology, Graduate School of Pharmaceutical Sciences, The University of Tokyo, Tokyo 113-0033, Japan; ari.ogaki@gmail.com (A.O.); yuji@ikegaya.jp (Y.I.); 2 Institute for AI and Beyond, The University of Tokyo, Tokyo 113-0033, Japan; 3 Center for Information and Neural Networks, National Institute of Information and Communications Technology, Suita City 565-0871, Japan

**Keywords:** extracellular vesicles, astrocytes, miRNA, targeting mechanisms

## Abstract

Extracellular vesicles (EVs) are composed of lipid bilayer membranes and contain various molecules, such as mRNA and microRNA (miRNA), that regulate the functions of the recipient cell. Recent studies have reported the importance of EV-mediated intercellular communication in the brain. The brain contains several types of cells, including neurons and glial cells. Among them, astrocytes are the most abundant glial cells in the mammalian brain and play a wide range of roles, from structural maintenance of the brain to regulation of neurotransmission. Furthermore, since astrocytes can take up EVs, it is possible that EVs originating from inside and outside the brain affect astrocyte function, which in turn affects brain function. However, it has not been fully clarified whether the specific targeting mechanism of EVs to astrocytes as recipient cells exists. In recent years, EVs have attracted attention as a cell-targeted therapeutic approach in various organs, and elucidation of the targeting mechanism of EVs to astrocytes may pave the way for new therapies for brain diseases. In this review, we focus on EVs in the brain that affect astrocyte function and discuss the targeting mechanism of EVs to astrocytes.

## 1. Introduction

Extracellular vesicles (EVs) are defined by the International Society for Extracellular Vesicles (ISEV) as "a general term for particles that are spontaneously released from cells and cannot be replicated, separated by a lipid bilayer" [1]. EVs can be classified into several categories, such as exosomes, microvesicles, and apoptotic vesicles, in terms of their properties and production mechanisms. However, many of the definitions on which these classifications are based are ambiguous in terms of size and synthetic pathways, and the ISEV recommended that they be used uniformly as "EVs" without distinction [2].

In 2007, Valadi and colleagues first suggested that EVs from mouse and human mast cells contain mRNAs and microRNAs (miRNAs) [3]. They also reported that mRNAs containing ^3^H-labeled uracil are translated by transferring them from donor cells (mouse mast cells; MC/9) to recipient cells (mouse mast cells; MC/9 and human mast cells; HMC-1) [3]. MiRNA contained in EVs have been suggested to affect the function of the recipient cells. MiRNAs in EVs derived from B cells infected with Epstein Barr virus (EBV) suppressed the expression of fractalkine CXCL11 in recipient cells (monocyte-derived dendritic cells) [4]. Another study suggested that miRNAs in EVs derived from hepatocellular carcinoma (Hep3B) suppressed the expression of transforming growth factor β activated kinase-1 (TAK1) in recipient cells (other Hep3B cells) [5]. In addition, it has been reported that cancer cell-derived exosomes increase the expression of mesenchymal-epithelial transition factor (MET) in bone marrow cells and promote angiogenesis in malignant melanoma [6]. These findings suggest that the effects of EVs are not limited to changes in gene and protein expression at the single recipient cell level and that EVs may affect the expression of biological functions through the regulation of intercellular communication.

Research on EVs has developed mainly in cancer research, but in recent years, the function of EVs in the central nervous system (CNS) has attracted much attention. EVs have been reported to be produced and secreted from cultured mouse and rat neurons [7] and cultured chicken, rat, and mouse astrocytes [8,9,10]. EVs have also been reported to functionally modulate synaptic plasticity and immune responses by regulating protein expression in recipient cells. Furthermore, it has been reported that EVs containing miRNAs are present in the human postmortem brain [11]. Thus, EV research has coalesced around the elucidation of donor cells, contents, and functions. However, due to the difficulties of identifying EV recipient cells, the targeting mechanisms of EVs to recipient cells are not well understood. Given that EVs can alter protein expression in recipient cells, it is likely that EVs are taken up in a cell type-specific manner rather than in a cell type-nonspecific manner, but the mechanisms are largely unknown. Since EVs have been widely studied for their potential role as cargo in drug delivery systems [12,13,14], it is important to clarify the targeting mechanisms to avoid adverse reactions [12,13,14].

**Figure 1:**
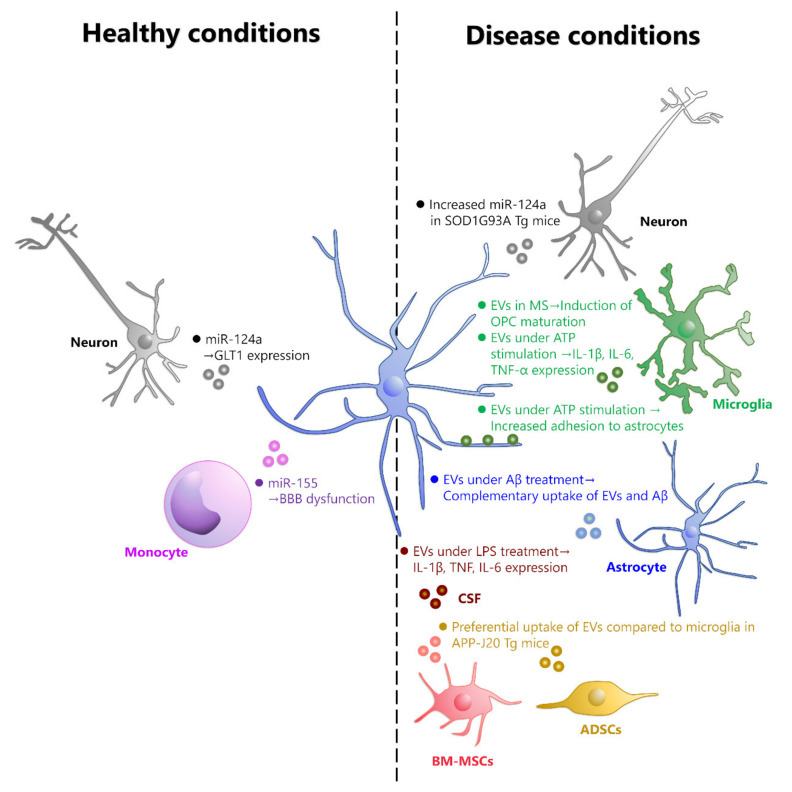
**EVs taken up by astrocytes under healthy and disease conditions.** EVs that are taken up by astrocytes under healthy conditions are mostly derived from neurons and monocytes. Neuronal-derived EVs contain miR-124a, which increases the expression of GLT1 in astrocytes. Monocyte-derived EVs contain miR-155, which can cause dysfunction of BBB. On the other hand, EVs that are taken up by astrocytes in disease conditions include those derived from neurons, microglia, astrocytes, adipose-derived stem cells (ADSCs), bone marrow-derived mesenchymal stem cells (BM-MSCs), and those contained in cerebrospinal fluid (CSF), although the cell type of origin is unknown. In SOD1G93A mutant mice, neuron-derived EVs contain high levels of miR-155 and are taken up by astrocytes. In MS model mice, astrocytes that have taken up microglia-derived EVs contribute to the maturation of OPCs. EVs from Aβ-treated astrocytes are taken up by astrocytes in a complementary manner to Aβ_1-42_. In hAPP-J20 Tg mice, EVs derived from ADSCs and BM-MSCs are taken up by astrocytes instead of microglia. In mice treated with LPS, EVs in CSF are taken up by astrocytes and increase the expression of IL-1β, TNF, and IL-6.

Astrocytes are the most abundant glial cells in the brain [15]. Astrocytes are involved not only in neuronal support, such as synapse formation and removal under healthy conditions [16,17] but also in the onset and exacerbation of CNS diseases including traumatic brain injury and spinal cord injury. In particular, aquaporin-4 (AQP4), expressed in astrocytes, is known to be involved in the pathogenesis of brain edema and has attracted attention as a therapeutic target [18,19]. Therefore, focusing on EV uptake by astrocytes may be useful in the search for therapeutic targets for brain diseases. However, most of the studies to date have been conducted under conditions that facilitate EV uptake, such as treating EVs with isolated astrocytes in a culture system. Therefore, there is little information on the conditions under which EVs are selectively taken up by astrocytes in vivo. In this review, we will discuss the effects of EVs on astrocytes in healthy and diseased brains and the possibility of astrocyte-specific targeting of EVs (Figure 1).

## 2. EVs That Are Taken up by Astrocytes under Healthy Conditions

Previous studies on EV-mediated intercellular communication have mainly focused on the secretion and function of EVs during disease. Therefore, the importance of EV-mediated regulation of cellular functions has been discussed in the context of disease. Recently, however, it has become clear that EVs are also secreted in the healthy brain. For example, it has been shown that EVs derived from brain cells exist in the peripheral blood of cynomolgus monkeys [20]. Furthermore, EVs are reported to be secreted by almost all major brain cells, including neurons [7], astrocytes [10], oligodendrocytes [21], and microglia [22]. In addition, EVs have been suggested to be functional in the healthy brain: EVs secreted by neurons during development are required for neuronal proliferation, differentiation, and the formation of neural circuits such as synapse formation and synchronous firing [23]. In the adult brain, neuron-derived EVs containing miR-132 are taken up by vascular endothelial cells and regulate the expression of vascular endothelial cadherin (VE-cadherin) [24].

In the following sections, we will summarize the uptake of EVs by astrocytes under healthy conditions, divided into different types of cells that serve as EV donors.

### 2.1. Uptake of Neuron-Derived EVs by Astrocytes

EVs derived from neurons in healthy conditions are possibly taken up by astrocytes and affect their function. It was reported that EVs derived from cultured mouse neurons contain miR-124a, which increases the expression of glutamate transporter 1 (GLT1) protein in astrocytes when EVs are taken up by cultured mouse astrocytes [25]. Thus, neurons may increase glutamate uptake by astrocytes via EVs, which may serve to maintain homeostasis of glutamate concentrations in the synaptic cleft. However, since the above study used cultured astrocytes as recipient cells for EVs, it remained unclear whether neuron-derived EVs would actually be taken up specifically by astrocytes specifically in the brain. Subsequently, it was also shown in vivo that miR-124a is taken up by astrocytes via neuron-derived EVs. Men et al. expressed GFP on neuron-specific CD63, which is an EV marker protein present on the membrane surface of EVs, under the calcium/calmodulin-dependent protein kinase II (CaMKII) promoter. In this paper, they found that neuron-derived EVs are also taken up by astrocytes in vivo [26]. However, it was reported that miR-124a is also present in microglia in vivo [27]. The source of miR-124a in microglia is unclear, but its presence implies that the phenomenon of neuron-derived EV uptake is not astrocyte-specific.

### 2.2. Uptake of Oligodendrocyte-Derived EVs by Astrocytes

There is no report showing that oligodendrocyte-derived EVs are specifically taken up by astrocytes under normal conditions. When EVs derived from cultured mouse oligodendrocytes were applied to mixed brain cell cultures (including neurons, oligodendrocytes, microglia, and astrocytes), EVs are taken up by more than 90% of microglia and approximately 20% of neurons, while they were hardly taken up by oligodendrocytes and astrocytes [28]. The mechanisms by which oligodendrocyte-derived EVs are selectively taken up by microglia have been reported. Phosphatidylserine (PS) expressed on the membrane surface of oligodendrocyte-derived EVs acts as an “eat-me” signal and is recognized by microglia, leading to micropinocytosis [29].

### 2.3. Uptake of Microglia- or Astrocyte-Derived EVs by Astrocytes

There is also no report showing that microglia- or astrocyte-derived EVs are taken up by astrocytes under healthy conditions. Most studies of microglia-derived EVs applied to cultured neurons as recipient cells to examine the effects of EVs on neurons [30,31]. Similarly, the effects of EVs secreted from astrocytes have been examined in neuronal cultures [32,33]. Further studies are required to conclude whether microglia- or astrocyte-derived EVs can be taken up by astrocytes and alter their function.

### 2.4. Uptake of EVs Derived from Peripheral Blood by Astrocytes

It has been shown that EVs in peripheral blood can pass through the blood–brain barrier (BBB) [34,35]. Furthermore, it has been reported that neuron-derived EVs are taken up by endothelial cells and that the expression of endothelial cadherin is increased by miR132 contained in EVs [24], suggesting that neuronal EVs may be involved in the regulation of BBB integrity. Therefore, EVs in the brain are derived from not only brain cells but also peripheral cells in the peripheral blood.

Among EVs in the peripheral blood under normal conditions, EVs derived from monocytes can be taken up by astrocytes. Cultured human monocyte-derived EVs have been shown to be taken up not only by cultured human neurons but also by cultured human astrocytes, and importantly, these EVs may contain miR-155, which is involved in increased permeability of the BBB [36,37]. Therefore, it is suggested that EVs from peripheral blood may affect CNS, including the possibility of increased infiltration of peripheral cells, such as leukocytes, due to uptake of EVs from peripheral organs by astrocytes and dysfunction of BBB integrity. Although it is necessary to consider EVs from peripheral organs, there are no other reports of EVs derived from peripheral blood under healthy conditions being taken up by astrocytes.

On the other hand, it has been reported that when EVs in mouse serum are intravenously administered to other mice, 86% of the EVs are taken up by microglia, and the rest are taken up by neurons, with almost no uptake by astrocytes [38]. Considering that EVs in this serum were observed mainly in ependymal cells of the third and lateral ventricles, it was likely that they crossed the blood-cerebrospinal fluid barrier (BCSFB) and entered the brain parenchyma. Although the cell types of EV sources remained unknown in Li and colleagues’ work, they assumed that the main source of secretion is likely to be peripheral monocytes and macrophages, since EVs derived from them were selectively taken up by microglia. Thus, there is a possibility that the uptake pathway of EVs varies depending on the cell type of EV source.

## 3. EVs That Are Taken up by Astrocytes under Disease Conditions

It has been reported that EVs contribute to disease progression or recovery in various brain diseases. Tau aggregation is one of the pathogeneses of Alzheimer’s disease (AD), in which early propagation of tau from the entorhinal cortex to the hippocampus occurs via microglia-derived EVs, which are involved in the progression of tauopathy [39]. In addition, aggregation and accumulation of α-synuclein is one of the pathologies of Parkinson’s disease (PD), and EVs derived from neurons are known to mediate this aggregation [40]. On the other hand, it has also been reported that the cellular prion protein (PrP^c^) on EVs derived from neuroblastoma cell types (N2a, SH-SY5Y) act in a neuroprotective manner by trapping amyloid-β (Aβ) and promoting fibrillation [41]. EVs derived from brain cells enter the peripheral blood in neurodegenerative diseases such as AD and PD; thus, their potential use as biomarkers of these diseases has been explored [42,43,44,45].

### 3.1. Uptake of Neuron-Derived EVs by Astrocytes

In the SOD1G93A transgenic mouse model of amyotrophic lateral sclerosis (ALS), when CD63 was labeled with GFP and miR-124a was labeled with Cy5 under the CaMKII promoter, the number of GFP puncta was not changed, but the colocalization ratio of GFP and Cy5 increased [46]. Taken these results and the fact that the SOD1G93A mutation did not affect miR-124a transcription together, it is suggested that the SOD1G93A mutation may increase the packaging of miR-124a by EVs. As mentioned above, it has been reported that miR-124a is taken up by astrocytes via neuron-derived EVs and alters the expression level of GLT1 in astrocytes [25]. Furthermore, it was reported that miR-124a was mostly transcribed in neurons and rarely in astrocytes [26]. While it has been reported that miR-124a expression in the cervical spinal cord is decreased in SOD1G93A mutant mice at the end stage of ALS [25], there has been no report on whether the targeting of EVs to astrocytes is altered. Considering that the expression level of GLT1 in astrocytes is decreased in ALS patients due to dysfunction of GLT1 splicing [47], it is possible that these changes may be caused by changes in the packaging of EVs and targeting astrocytes.

### 3.2. Uptake of Oligodendrocyte-Derived EVs by Astrocytes

As mentioned above, it has been suggested that EVs derived from oligodendrocytes may be selectively taken up by microglia. To date, there is no report that this selectivity is altered in brain diseases.

### 3.3. EVs Derived from Microglia

EVs released from cultured rat microglia could be taken up by astrocytes when cultures were treated with a cocktail of inflammatory cytokines, IL-1β, TNF-α, and IFN-γ [48]. Lombardi et al. suggested that upon exposure to these inflammatory cytokines in multiple sclerosis, microglia-derived EVs may be involved in myelin regeneration by astrocytes. When microglia-derived EVs were applied to cultured oligodendrocyte precursor cells (OPCs) alone or OPCs and astrocyte coculture, maturation of OPCs was inhibited only in the coculture of OPCs and astrocytes. OPCs differentiate into mature oligodendrocytes and form myelin. Therefore, it is possible that the maturation of OPCs is promoted as a result of the action of EVs on astrocytes rather than the direct action of EVs on OPCs.

ATP is released from dead cells into the extracellular space under injury and inflammatory conditions [49]. Mimicking this situation, it has been reported that ATP-stimulated microglia-derived EVs (ATP-EVs) are released from cultured microglia stimulated by extracellular ATP, resulting in the uptake of ATP-EVs by astrocytes [50]. The ATP-EVs were kept in contact with astrocytes using the IR laser of the optical tweezers for 30 s, then the IR laser was shut down, and the subsequent interaction between EVs and astrocytes was observed. The results showed that the contact time with astrocytes was longer with ATP-EVs than with control EVs [50]. Although ATP-EVs are thought to be taken up by astrocytes soon after contact, the expression of inflammatory cytokines, such as IL-1β, IL-6, and TNF-α, is upregulated in astrocytes after 48 h of ATP-EV treatment [50]. In addition, inhibition of PS-mediated contact between ATP-EVs and astrocytes by Annexin-V did not result in an increase in astrocytic proinflammatory cytokines [50]. Furthermore, treatment of astrocytes with isolated lipid membranes of ATP-EVs alone did not result in such an increase in proinflammatory cytokines [50]. Therefore, these results suggested that internalization of cargo after contact of ATP-EVs with astrocytes induces altered expression of astrocytes. Given the secretion of these cytokines from astrocytes and the possible increase in EV uptake by astrocytes with these cytokines, it is also possible that EVs derived from microglia taken up by astrocytes may further increase in vivo.

### 3.4. Uptake of Astrocyte-Derived EVs by Astrocytes

Aβ induced the release of EVs from cultured astrocytes [51]. It is also known that Aβ is produced during the aberrant processing of amyloid precursor protein (APP), which is involved in the pathogenesis of AD, and that this processing occurs in early endosomes and that Aβ is internalized into EVs [52]. It has also been suggested that Aβ and EVs share the uptake pathway. When EVs derived from cultured mouse astrocytes in addition to Aβ1–42 (composed of 42 amino acid residues with high aggregation capacity and neurotoxicity) were treated with cocultures of microglia and astrocytes, both EVs and Aβ1–42 were taken up by both microglia and astrocytes [53]. However, the percentage of EV uptake decreased in the presence of Aβ1–42, suggesting that EVs may be taken up in a complementary manner with Aβ1–42, which suggests that EVs and Aβ1–42 share the uptake pathway.

### 3.5. EVs Derived from Other Cell Types

It has been reported that EVs in peripheral blood in neurodegenerative diseases are taken up by brain cells, mostly microglia. When EVs in plasma from PD patients were administered to the mouse striatum, they were taken up by microglia but not astrocytes or neurons [54]. Xia and colleagues also showed they were involved in the inhibition of autophagy and accumulation of α-synuclein [54]. Although the origin of EVs in peripheral blood in this study is not clear, EVs are produced by adipose-derived stem cells (ADSCs) [55] and secreted into the blood [56].

In a mouse model of AD using hAPP-J20 mice with long-term overproduction of Aβ, intrahippocampal administration of peripheral blood-derived EVs decreased the rate of microglial uptake of EVs, although most of the EVs in wild-type mice were specifically taken up by microglia [57]. In this study, it remains unclear the origin of EVs and whether EVs that were not taken up by microglia were subsequently taken up by astrocytes or neurons. Since EVs derived from ADSCs and bone marrow-derived mesenchymal stem cells (BM-MSCs) are secreted in AD models [58], it is possible that these cell types are the origin of EVs. In addition, if Aβ and peripheral blood-derived EVs are also taken up in a complementary manner, it is possible that EV uptake by microglia is suppressed by Aβ. It is also possible that there is compensatory uptake of residual EVs in the brain parenchyma by other cell types, such as astrocytes.

Balusu et al. isolated EVs from the cerebrospinal fluid (CSF) of mice treated with lipopolysaccharide (LPS) to induce inflammation and inject EVs into the ventricle [59]. They showed that EVs are uptaken by both astrocytes and microglia but not neurons. Furthermore, when EVs were applied to mixed cortical cultures (including astrocytes, microglia, and neurons), inflammation-related gene expression, such as IL-1β, TNF, and IL-6, was upregulated in the supernatant. Thus, EVs derived from outside the brain may also be taken up by brain cells, including astrocytes, and work functionally.

Transcytosis by choroid plexus ependymal cells may be a possible mechanism for the intracerebral transfer of peripheral EVs. Folic acid, a water-soluble vitamin, needs to be transferred from the periphery to the brain, and EVs are involved in this transfer [60]. Folic acid is converted to its biologically active form, 5-methyltetrahydrofolate (5MTHF), through various enzymatic reactions. It is then endocytosed from the bloodstream via folate receptor-α (FR-α) in choroid plexus epithelial cells, internalized by EVs expressing FR-α on their membrane surface, and discharged into the CSF. Subsequently, FR-α-positive EVs pass through ependymal cells and are predominantly taken up by astrocytes, while most FR-α-negative EVs do not enter the brain parenchyma and are taken up by ependymal cells [60]. Considering that 36% of the EVs present in human CSF express FR-α [60] and that the choroid plexus expresses FR-α in the brain [61], it is possible that the choroid plexus is a major source of EVs in CSF.

Furthermore, it has been reported that EVs can be transferred in both directions, central to peripheral and peripheral to central, and that EVs in the brain are contained in peripheral blood [42,62,63]. The route that EVs take during this transition remains unresolved, but a BBB- or BCSFB-mediated route has been suggested [64,65]. Since BBB dysfunction occurs in various brain diseases [66], it is possible that the brain trafficking of EVs is affected depending on the disease. Therefore, the origin of EVs in peripheral blood may be brain cells as well as peripheral tissues, and further studies are needed to distinguish the cell types from which EVs are derived.

## 4. The Possible Mechanisms of EV Targeting to Astrocytes

In this review, we discussed the possibility that EVs from different cellular sources can be taken up by astrocytes under conditions of health and disease (Figure 1). However, the method of EV purification needs to be carefully considered. This is because purification of EVs is difficult and various materials are likely to contaminate along with EVs, including vesicle-free miRNAs and protein aggregates [67]. Precipitation-based exosome isolation kits combined with filtration have been successful in reducing the number of vesicle-free miRNAs but are not suitable for small volume purification [68]. Thus, it is still difficult to purify completely contamination-free EVs. Therefore, the effects of contamination must always be considered in functional experiments with purified EVs.

Various mechanisms have been proposed for the uptake of EVs, including membrane fusion, macropinocytosis, phagocytosis, and endocytosis (clathrin-dependent, lipid raft-dependent, and caveolin-dependent) [69,70]. Previous studies have used pharmacological inhibitors to elucidate the mechanisms of EV uptake. Among them, the only mechanism of EV uptake in CNS that has been clarified is microglial uptake, which is a pathway mediated by macropinocytosis [29,71]. For example, PS exposed on the EV surface has been shown to be a targeting mechanism of EVs to microglia [29]. Many of the currently reported EVs are predominantly taken up by microglia, and the mechanisms of EV uptake into brain cells other than microglia remain largely unknown. This is reasonable given that microglia are resident macrophages in the brain [72]. Therefore, in this section, we will introduce the EV targeting mechanism to microglia and discuss the possible EV targeting mechanism in astrocytes.

Astrocytes have been reported to have the ability of pinocytosis [73,74]; thus, if macropinocytosis is the main uptake pathway, selective uptake by microglia is unlikely. Although macropinocytosis-mediated uptake of EVs into microglia has been shown thus far, phagocytosis-mediated uptake has not been completely ruled out. On the other hand, microglial and astrocytic phagocytosis can be cooperative. When microglial phagocytosis is impaired, astrocytes compensate for phagocytosis by activating Mertk, a member of the Tyro3/Axl/Mertk (TAM) family [75]. Mertk is expressed not only in microglia but also in astrocytes [76,77]. Since TAM receptors are involved in phagocytosis by recognizing PS exposed on the membrane surface [78], it is possible that astrocytic phagocytosis of PS-labeled EVs serves as a compensatory mechanism when the phagocytic capacity of microglia is reduced. If EV uptake is mediated by macropinocytosis or phagocytosis, then in principle, both astrocytes and microglia should be capable of EV uptake. However, in reality, many reports indicate that microglia selectively take up EVs, and the reasons for this may be differences in brain regions and experimental timings. For example, it is known that TAM receptors are required for synaptic pruning of the visual system by astrocytes during development but not by microglia [79].

It is also possible that EVs are not taken up by astrocytes in exactly the same way as microglia. For example, cell adhesion molecules may play a role because the behavior of ATP-EVs, such as the number of contacts and movements between microglia and astrocyte surfaces, as well as the frequency of internalization, are different [80]. Furthermore, prolonged contact time of ATP-EVs with astrocytes was reported [50]. Although the responsive proteins for the above phenomena are unknown, proteomic analysis of ATP-EVs showed a nearly twofold increase in GO biological process terms related to cell adhesion compared to control EVs [50]. KEGG analysis also showed a significant increase in protein expression in gap junctions, focal adhesion, and adhesion binding [50]. Since the protein represented by GO:0007155 was increased in ATP-EVs in particular, analysis focusing on these 3189 proteins may elucidate the targeting mechanisms of EVs to astrocytes.

Thus, the mechanisms of EV uptake by astrocytes leave much to be elucidated. One reason for this is that EV uptake by astrocytes is a relatively rare phenomenon, especially in the healthy brain, where astrocytes may be the cells responsible for secretion rather than uptake of EVs [81]. Another main reason is that many studies have treated cultured cells with isolated and labeled EVs and observed uptake but have not compared EV uptake between other brain cell types and astrocytes. This makes it difficult to elucidate cell type-specific or non-specific targeting mechanisms. The best way to solve this problem would be to administer pre-labeled EVs in vivo, but this method has limitations in applying live imaging and pharmacological validation, which play an important role in elucidating the EV uptake mechanism. Therefore, humanized self-organized models, organoids, 3D cultures and human microvessel-on-a-chip platforms are useful to compare EV uptake [82,83,84]. These in vitro systems can maintain the spatial location of the cells, allowing us to verify EV uptake mechanisms that are closer to in vivo. We believe that these methods will contribute to the elucidation of the targeting mechanisms of EVs to astrocytes.

## 5. Conclusions

Few studies have examined the potential of astrocytes as EV recipient cells. Comparison of the EV uptake mechanism with that of microglia, which have a high EV uptake capacity, will help to elucidate the targeting mechanism of EVs to astrocytes. Using organoids or 3D culture systems and proteomic analysis of EVs with particularly high uptake by astrocytes will also help to elucidate the targeting mechanism. In addition, the effect of EVs taken up by astrocytes on astrocyte function has not been fully elucidated, and further research is needed. Clarification of the astrocyte-specific EV uptake mechanism and its effects will shed light on the establishment of new therapies for brain diseases using astrocytes, which are the most abundant glial cells in the brain.
